# Supplementary material for: Content-rich biological network constructed by mining PubMed abstracts
Source: BMC Bioinformatics. 2004 Oct 8;5:147. doi: 10.1186/1471-2105-5-147 (PMC528731; doi:10.1186/1471-2105-5-147)
Supplement: Additional File 2 — The original results of the above study (non-essential files are deleted to keep the file size under the limit set by BMC bioinformatics). [file 1471-2105-5-147-S2.bz2 › chilibotAdditionalFile2/dip05/109ID7589433E448/html/PIK3R1_IGF1R.html]

 


 **PIK3R1** and **IGF1R** 
  
Found 11 abstracts in PubMed, retrieved 05.  
 

 What does Google say? 
 PDF only 
| .edu only 

---

- J NutrJ Nutr,   **Conjugated Linoleic Acid Downregulates Insulin Like Growth Factor I Receptor **[IGF1R]** Levels in HT 29 Human Colon Cancer Cells.** .
  Conjugated linoleic acid CLA has chemoprotective properties in a variety of experimental cancer models.
  We have previously observed that dietary CLA inhibits colon tumorigenesis induced by dimethylhydrazine in rats.
  In addition, our in vitro studies have shown that CLA inhibits DNA synthesis and induces apoptosis in HT 29 cells, the human colon adenocarcinoma cell line.
  The insulin like growth factor IGF system regulates the growth of HT 29 cells by an autocrine mechanism.
  The present study examined whether the growth inhibitory effect of CLA is related to changes in the IGF system in HT 29 cells.
  To determine whether CLA inhibits IGF II production, HT 29 cells were incubated in serum free medium in the presence of various concentrations of CLA.
  CLA decreased protein levels of both mature and pro IGF II and IGF II transcripts.
  Whereas exogenous IGF I and IGF II produced an increase in cell number, neither IGF I nor IGF II counteracted the negative growth regulatory effect of CLA.
  Reverse transcriptase polymerase chain reaction and Western blot analysis of total cell lysates revealed that CLA decreased IGF I receptor IGF IR transcript and protein levels in a dose dependent manner.
  Immunoprecipitation Western blot studies revealed that CLA inhibited IGF I induced phosphorylation of IGF IR and insulin receptor substrate IRS 1, recruitment of the p85 regulatory subunit of phosphatidylinositol 3 kinase PI3K **[PIK3R1]** to IGF IR, IGF IR associated PI3K **[PIK3R1]** activity, and phosphorylated Akt and extracellular signal regulated kinase ERK 1 2 levels.
  In conclusion, the inhibition of cell proliferation and induction of apoptosis by CLA in HT 29 cells may be mediated in part by its ability to decrease IGF II synthesis and to downregulate IGF IR signaling and the PI3K **[PIK3R1]** Akt and ERK 1 2 pathways.

  - Recent Prog Horm Res, 2003   **Microarray analysis and identification of novel molecules involved in insulin like growth factor 1 receptor **[IGF1R]** signaling and gene expression.** .
    The insulin receptor IR and the insulin like growth factor 1 receptor **[IGF1R]** IGF 1R are members of the same subfamily of receptor tyrosine kinases.
    The two receptors phosphorylate many of the same substrates and activate the same signaling modules, including the mitogen activated protein kinase MAPK and phosphatidyl inositol 3 kinase PI3K **[PIK3R1]** signaling pathways.
    Although the IR and IGF 1R share some redundant functions in metabolism, cell growth, differentiation, and apoptosis, they also exhibit distinct physiological roles.
    Some of these may be due to differences in tissue distribution, receptor structure, formation of hybrid receptors, or mechanisms of ligand binding.
    However, the divergent effects of insulin and IGF 1 also may be explained by specificity in the intracellular signals generated by insulin and IGF 1.
    In particular, the IR and IGF 1R are capable of triggering their own biological responses by using specific or preferential substrates, molecular adapters, or signaling pathways.
    In a recent study, we used cDNA microarray analysis to identify genes differentially regulated by insulin and IGF 1.
    Mouse NIH 3T3 fibroblasts expressing either the wild type human IGF 1R or IR were stimulated with either IGF 1 or insulin, respectively.
    We identified 39 genes differentially regulated by insulin and IGF 1.
    Most of these genes had not been reported previously to be responsive to insulin or IGF 1.
    The genes induced by IGF 1 generally were involved in mitogenesis or differentiation, while the genes found to be induced by insulin did not conform to any particular category.
    In a separate study, immortalized breast epithelial cells were stimulated with IGF 1 and a cDNA microarray analysis was used to generate a profile of IGF 1 regulated genes.
    A number of genes known to be involved in angiogenesis were found to be regulated by IGF 1.
    These results strongly suggest that this technology may be extremely useful in identifying groups of genes that are specifically regulated by different ligands and their activated receptors.

    - Diabetes, 2002   **Increased insulin sensitivity in IGF I receptor deficient brown adipocytes.** .
      Immortalized brown adipocyte cell lines have been generated from fetuses of mice deficient in the insulin like growth factor I receptor **[IGF1R]** gene IGF IR , as well as from fetuses of wild type mice IGF IR .
      These cell lines maintained the expression of adipogenic and thermogenic differentiation markers and show a multilocular fat droplets phenotype.
      IGF IR brown adipocytes lacked IGF IR protein expression.
      insulin receptor IR expression remained unchanged as compared with wild type cells.
      Insulin induced tyrosine autophosphorylation of the IR beta chain was augmented in IGF IR deficient cells.
      Upon insulin stimulation, tyrosine phosphorylation of insulin receptor substrate 1 IRS 1 was much higher in IGF IR brown adipocytes, although IRS 1 protein content was reduced.
      In contrast, tyrosine phosphorylation of IRS 2 decreased in IGF IR deficient cells.
      its protein content was unchanged as compared with wild type cells.
      Downstream, the association IRS 1 growth factor receptor binding protein 2 Grb **[PIK3R1]** 2 was augmented in the IGF IR brown adipocyte cell line.
      However, SHC expression and SHC tyrosine phosphorylation and its association with Grb **[PIK3R1]** 2 were unaltered in response to insulin in IGF IR deficient brown adipocytes.
      These cells also showed an enhanced activation of mitogen activated protein kinase MAPK kinase MEK1 2 and p42 p44 mitogen activated protein kinase MAPK upon insulin stimulation.
      In addition, the lack of IGF IR in brown adipocytes resulted in a higher mitogenic response DNA synthesis, cell number, and proliferating cell nuclear antigen expression to insulin than wild type cells.
      Finally, cells lacking IGF IR showed a much lower association between IR or IRS 1 and phosphotyrosine phosphatase 1B PTP1B and also a decreased PTP1B activity upon insulin stimulation.
      However, PTP1B Grb **[PIK3R1]** 2 association remained unchanged in both cell types, regardless of insulin stimulation.
      Data presented here provide strong evidence that IGF IR deficient brown adipocytes show an increased insulin sensitivity via IRS 1 Grb **[PIK3R1]** 2 MAPK, resulting in an increased mitogenesis in response to insulin.

      - Oncogene, 2001   **Aberrant expression and activation of insulin like growth factor 1 receptor **[IGF1R]** IGF 1R are mediated by an induction of IGF 1R promoter activity and stabilization of IGF 1R mRNA and contributes to growth factor independence and increased survival of the pancreatic cancer cell line MIA PaCa 2.** .
        In the present study we investigated the mechanisms responsible for and the biological consequences of the constitutive activation of the insulin like growth factor 1 receptor **[IGF1R]** IGF 1R in the MIA PaCa 2 cells.
        An aberrant increase in the expression and activation of the IGF 1R was observed during the transition of growth states from exponential to quiescent.
        The increase in IGF 1R expression is preceded by an increase in IGF 1R mRNA transcript and is associated with an increase in the IGF 1R promoter activity.
        Inhibition of de novo transcription by actinomycin D increased the stability of IGF 1R mRNA in exponentially growing cells, thereby increasing the expression of IGF 1R to a level similar to that seen in quiescent cells.
        Increased IGF 1R signaling mediated the growth factor independence of quiescent MIA PaCa 2 cells through the constitutive activation of mitogen activated protein kinase MAPK .
        Exogenous IGF 1 increased cell proliferation and activated MAPK and AKT signaling pathways.
        The resistance of cells to apoptosis by IGF 1R signaling was mediated through MAPK and phosphatidylinositol 3 kinase PI3K **[PIK3R1]** pathways and a yet unidentified pathway s .
        Thus, aberrant regulation of IGF 1R signaling is required for resistance to apoptosis and growth factor independence of MIA PaCa 2 cells.
        This likely protects cells from unfavorable conditions and allows cells to rapidly re enter the cell cycle when conditions are favorable.
